# Observations on the Occasional Injurious Effects of Leeches

**Published:** 1808-01

**Authors:** James Stuart


### *Observations on the occasional injurious Effects of Leeches.* By James Stuart, M.D.

PHYSICIANS observing the bites of leeches to be occasionally followed by troublesome inflammation, ulceration, and gangrene, have attributed these to some venomous quality of the insect. But, as the precautions founded on this opinion have proved ineffectual in obviating the evils noticed, the opinion may be justly suspected to be fallacious.

Though the species of the leech be considerably numerous, there are but three which will stick. The snail tailed, the broad tailed, and the large brown leech with a reddish belly. The first adheres tolerably well; but growing only to an inch in length, they cannot be employed to any advantage. The second grows to an inch and a half; but from an uncertainty in his functions, always gives place to the large brown leech, which last is, at this time, the species most generally employed in medicine. This, in in common with the same family, has the general figure of the worm, and grows from two to four inches long. The body is composed of rings, which supply the power of contraction and of swimming. The skin on the back is blackish, or brown, bespangled with minute yellow spots, which, as it approaches the sides, is bordered with a narrow streak of yellow. From this last, the belly is of a reddish colour, and interspersed with a number of yellow spots. His head is small. But that which claims our attention most particularly, is the structure of his mouth. This is of a triangular figure, and composed of two lips, which are made to conform at pleasure, to the convenience of the animal. Within these are situated three solid teeth, so sharp and strong, that they are capable of piercing the skins of men, or even those of horses or oxen. A little posterior, is observed a small protuberance, answering, in appearance, and most probably in use, to a tongue, which, when flaccid, very exactly closes all the cavity within the teeth. With this structure of the leech, his thirst of blood is proverbial. Hence Horace,

> Quem vero arripuit, tenet occiditque legendo,
> *Non missura cutem nisi plena cruoris hirudo.*

Whether accident or design has presented him with his prey, the first care of the leech is to prepare an opening for the exit of his favourite repast. This is done by inserting his three fangs into the skin. Next, withdrawing these, he closes his lips round the wounds and retracts his tongue farther into his mouth, so as to produce a vacuum. By which means, the weight of the atmosphere being removed from the blood-vessels embraced by his lips, while its force remains the same on the parts without, the blood is forced into the mouth so strongly, as to fill his twenty-four little stomachs, containing in the aggregate from two to three drachms of blood, in the short space of a few minutes.

From what has been said, it appears there is but one species of leech commonly employed for medical pnrposes. From hence it follows, the opinion that the bad effects from their bites proceed from the quality of the leech, must be erroneous. For, this being admitted, to suppose these are induced by a venomous quality, is to suppose, that some individuals are endowed with a quality with which others of the same species of animals are not; which would be absurd. I am aware, it may be said that this noxious quality of the leech is adventitious, and that it depends upon the qualities of the waters from which they they are taken. But this supposition, still, does not solve the difficulty; for as they are all kept a considerable time before use, in clean water, and this often changed, their supposed poisonous qualities, were they adventitious, must be washed off, and we, consequently, should never witness any bad effects from their bites.

The fallacy of the generally received opinion, further appears from the organization of the insect. For, as the teeth are destitute of punctures in their points, they are not calculated to the introduction of any substance into a wound. And, were it even possible any poisonous particles should adhere to the teeth, and be introduced with them, as the animal feeds by suction, its very functions, joined to the constant efflux of blood from the punctures, must prove a certain preventive to these particles remaining long enough to produce any mischievous effects.

Much more might be said, but as this is deemed sufficient to prove that injurious consequences from the bites of leeches cannot depend upon any innate or adventitious quality in them, I shall dismiss the subject, and proceed to inquire, on what these consequences do depend.

I attribute them to the state of the system, or to that of the particular part to which they are applied.

I conclude this to be the case,

1. Because the effects mentioned never occur, except in such cases as evidence the presence of a morbid diathesis, general or local, in the part to which they are applied.

2. Because they frequently do occur where such diathesis exists.

Of the truth of the first of these positions, we have daily proofs in the application of leeches in ophthalmia, where the disease is topical and they are not applied immediately to a part under inflammation. In this case, bad effects never succeed.

Cases of inflammation, ulceration, or gangrene, from the application of leeches in a general morbid diathesis, or immediately to a part under disease, have occurred under the observation of almost every practitioner who has ever been in habits of employing them.

I have witnessed many; the following of Capt. R. A. mentioned in Vol. I. No. 2, communication the 10th, of the Philadelphia Medical Museum, seems, to me, one of the most impressive, and well calculated to my purpose. In this case the diathesis had a strong tendency to gangrene.

When I first saw him, on the 31st of July last, a partial sphacelus sphacelus had taken place in one of the nates, and the cellular membrane of the perinæum was destroyed. The scrotum was nearly divested of cuticle, and much tumefied; but, as I understood, had subsided since the attendance of Dr. Rush. I was informed blisters had been applied to the scrotum, and to the parts affected with sphacelus on the 29th, when the Doctor was first called in; and leeches to the scrotum on the 30th. August 1st, there was an appearance of gangrene on the scrotum.—August 2d, appeared several sphacelated spots, the size of the finger nail, immediately *on the parts of it to which the leeches had been applied.* The same day they were punctured with a lancet. August 3d, the sphacelus had extended so far as to destroy nearly one half of it. On the same day, in concurrence with Dr. Rush, I removed with the knife, several large portions of sphacelated skin and cellular membrane from the nates and perinæum. On the 4th I laid open the perinæum, in its whole length, from near the anus to the scrotum, down to the urethra. The 6th I removed with the scalpel, *all that part of the scrotum to which the leeches had been applied,* being nearly one half, down to the testis.

The leech produces the injuries here noticed, by the formation of a vacuum on the part to which he is applied.—By this means, the usual support from the pressure of the atmosphere being removed from a part of the system, while it remains the same on other parts, the tone of the part under the influence of the leech is diminished, while, at the same time, and by the same causes, that of the latter is relatively increased.

And hence arise two most certain and active predisposing causes of inflammation, a general increase of tone and a local debility.

From this view of the subject, I conclude that bad consequences are to be apprehended from leeches.

1. When applied in an inflammatory diathesis before general bleedings have been performed.
2. In a general gangrenous diathesis.
3. In an extremely irritable habit.
4. And last, to a part actually affected with inflammation, ulceration, or gangrene.

